# The Role of Self-Efficacy, Task Value, and Intrinsic and Extrinsic Motivations in Students’ Feedback Engagement in English Learning

**DOI:** 10.3390/bs13050428

**Published:** 2023-05-19

**Authors:** Zhengdong Gan, Fulan Liu, Honghan Nang

**Affiliations:** 1 Faculty of Education, University of Macau, Macau 999078, China; 2 Foreign Language College, Jiangxi Normal University, Nanchang 330022, China; fulan.liu@yahoo.com; 3 Foreign Languages Department, China Pharmaceutical University, Nanjing 210009, China

**Keywords:** self-efficacy, task value beliefs, intrinsic and extrinsic motivations, multiple regression analyses

## Abstract

Drawing on Wigfield and Eccles’s motivational theory, which is acclaimed for explaining individual behavioral intentions, this study investigated the extent to which different forms of motivation (i.e., self-efficacy, task value, and intrinsic and extrinsic motivations) predicted student behavioral feedback engagement (i.e., action on teacher feedback and feedback seeking) in English learning. The participants were 276 male and female students who were enrolled in a second-year full-time English language and literature program at two Chinese universities. Multiple regression analyses showed that task value emerged as the only motivational variable that significantly predicted both students’ action on teacher feedback and feedback seeking. Intrinsic motivation significantly predicted action on teacher feedback, whereas extrinsic motivation and self-efficacy significantly predicted feedback seeking. Pedagogical implications for endeavors to support students in their engagement with feedback in learning English as a foreign language in China are discussed.

## 1. Introduction

While the benefits of feedback are frequently mentioned in articles about learning and teaching, a major challenge for productive feedback processes is the difficulty of generating student engagement with the feedback they receive, which may limit student learning opportunities and undermine their confidence and motivation, leading to disengagement and even withdrawal from the feedback processes [1]. Although the research literature on feedback in the field of instructed second-language acquisition has grown in recent years, a substantial body of such research has focused on the effects of different types of corrective feedback strategies on second-language development (e.g., [2,3,4]). This line of research has provided important insights into the contribution of corrective feedback to students’ developing second-language system.

Meanwhile, some researchers investigating second language feedback have explored individual learner differences underlying students’ preferences for different kinds of corrective feedback (e.g., [5,6,7]). The findings of these researchers’ studies highlight the importance of investigating the role of individual differences in the feedback processes. While these findings have offered insights into learner feedback training aimed at improving students’ noticeability and uptake of corrective feedback [3,8], an important and practical question for feedback researchers and practitioners remains: what motivates students to engage with feedback in the normal course of learning a second language? Moreover, researchers in general education and educational psychology are calling for a shift from the provision of corrective feedback (e.g., teacher evaluative feedback) to the design of learning environments and the seeding of productive learning tasks that result in learning process-oriented feedback, which in turn fosters self-regulation in students (e.g., [9,10]). It is in this context that the current study aimed to investigate the dynamics of students’ motivational processes (i.e., self-efficacy, task value, and intrinsic and extrinsic motivations) in relation to their feedback use and feedback-seeking experience (i.e., action on teacher feedback and feedback seeking) while they were studying in an academic English course at university.

Following prior research on self-regulated language learning (e.g., [11,12,13]), this study depicts students as having an essential role in feedback engagement, which is, viewed as a crucial element of self-regulation in educational psychology [14,15]. It is, therefore, important to examine the key motivational antecedents underlying students’ feedback engagement to inform pedagogy, particularly in a second-language learning context where students are often unlikely to attain a high level of target language competence unless they are capable of regulating their own learning behavior and taking responsibility for their learning (e.g., [12]). Consequently, this study differs from previous second-language feedback studies in that our focus is not confined to a particular form of feedback, such as written or oral feedback, and we look at student feedback engagement in the form of action on teacher feedback and feedback seeking across a tertiary-level English language and literature course. Specifically, drawing on the motivational theory proposed by Wigfield and Eccles [16], this study addressed the following two research questions: 

1. What are the relationships between four motivational variables (i.e., self-efficacy, task value, and intrinsic and extrinsic motivations) and student action on teacher feedback in the context of an English language and literature program at a Chinese university?

2. What are the relationships between four motivational variables (i.e., self-efficacy, task value, and intrinsic and extrinsic motivations) and student feedback seeking in the context of an English language and literature program at a Chinese university?

## 2. Expectancy–Value Theory

An important theoretical framework to understand individuals’ motivational beliefs and their learning behavior in the literature is the expectancy–value model [16,17]. From an expectancy–value theoretical perspective, motivation is typically defined as the processes that allow people to select appropriate goals and pursue them successfully [18]. Motivational factors are, therefore, considered to be part of an individual’s goal structures and beliefs about what is important [19]. Within the expectancy–value model of motivation, expectancy is defined as individuals’ beliefs about how well they will successfully perform a goal-oriented action. In other words, expectancy is concerned with learners’ competence-related beliefs in certain tasks [17]. Since Bandura’s [20] self-efficacy theory also deals with expectancies for success, expectancies and self-efficacy are thus often treated as the same general factor in the literature [21]. Within the expectancy–value theory, value refers to the worth attributed to the achievement, enjoyment, and usefulness of a task [17] and includes three components: attainment value or importance, intrinsic value, and utility value of the task. According to Wigfield and Eccles [16], intrinsic value is a construct related to the construct of intrinsic motivation as defined by Deci and his colleagues (e.g., [22,23,24]), whereas utility value can be tied to the construct of extrinsic motivation [23,24,25] as utility value captures more extrinsic reasons for engaging in a task, such as performing a task to reach a desired end state. In this study, we adopted the expectancy–value theory as the theoretical framework to examine the relations between four motivational variables (i.e., self-efficacy, task value, and intrinsic and extrinsic motivations) and two types of students’ behavioral feedback engagement (i.e., action on teacher feedback and feedback seeking).

In the Chinese culture, education is regarded as the path to success and meaningful participation in society throughout life [26]. Chinese students tend to believe that effort and perseverance are more important for success than ability [27], and they, therefore, exert much effort in academic work as they believe that success is the result of hard work. Furthermore, proficiency in English is regarded in China as a definite asset of considerable value, both at an individual and a societal level, that gives students access to the knowledge and skills they need to participate fully in the wider world in the era of economic globalization [28]. Previous research suggests that Chinese students tend to believe that they can reach a high level of proficiency in English through effort and perseverance [29]. Given the cultural and educational context in which this study was situated, the expectancy–value theory, which is acclaimed for explaining individual behavioral intentions, serves as a particularly useful frame of reference to help conceptualize motivational factors that might influence Chinese students’ feedback engagement in English learning.

### 2.1. Self-Efficacy

From a social cognitive theory perspective, self-efficacy represents people’s beliefs in their capabilities to organize and execute a given course of action to solve a problem or accomplish a task [20]. Self-efficacy is viewed as analogous to the expectancy construct in the expectancy–value theory [30]. Self-efficacy beliefs have been found to influence learners’ choice of achievement tasks, persistence on those tasks, vigor in carrying those tasks out, and performance on those tasks [16]. It is posited that “the stronger the sense of personal efficacy, the greater the perseverance and the higher the likelihood that the chosen activity will be performed successfully” ([14], p.314). For example, research shows that students with stronger self-efficacy beliefs persist longer when they encounter difficulties in English learning and tend to be more self-regulating in the use of strategies for studying [31]. Wang and Bai [32] also noted that self-efficacy beliefs significantly predicted self-regulated learning behaviors and English language test scores. These empirical findings have significant implications as knowledge of students’ perceived self-efficacy and its relation to other factors, such as self-regulated learning strategies, will allow teachers to know their students well and adjust their teaching practices in the classroom accordingly. 

### 2.2. Task Value

In the expectancy–value theory, task value refers to students’ perceptions of how important, how interesting, and how useful a particular task is. Task value, thus, differs from goal orientation in that goal orientation concerns the reasons why students are participating in a particular task [33], whereas task value concerns the value attributed to the importance, enjoyment, and usefulness of a task [34]. While these specific components of task value are presumed to be distinct from each other [35], a substantial body of research studies in the literature has shown that these value components are highly correlated and can be combined into a single composite measure. Like expectations of success, task values are postulated as significant proximal predictors of course performance and achievement in academic settings [36]. For example, Song and Chung [37] reported that task value was a strong predictor of achievement-related choice behaviors, persistence, and effort. In the present study, we were interested to look at how English learning-related task value might impact on students’ behavioral feedback engagement.

### 2.3. Intrinsic and Extrinsic Motivations

It is recognized in research that learners tend to work harder to achieve understanding and make greater progress when they are motivated to learn something [38]. Research into motivational dynamics in academic contexts based on the self-determination theory has centered largely around the intrinsic and extrinsic motives that regulate learners’ study behaviors [23,24,39]. According to the self-determination theoretical framework, the distinction between intrinsic motivation and extrinsic motivation relates to learners’ perceived reasons for their learning behaviors [40]. Intrinsic motivation usually represents undertaking a learning activity for its inherent interest and enjoyment; due to a preference for challenging academic work and the desire to explore and learn new things; or due to a preference for mastering academic material independently [41]. The distinctions between various types of extrinsic motivation depend on the extent to which people have been successful in internalizing the initially external regulation of the behavior [39]. For example, the least autonomous form of extrinsically motivated behavior represents a behavior prompted by external contingencies, such as rewards, punishments, and deadlines. Another common type of extrinsic motivation refers to the situation in which individuals have internalized a formerly external source of motivation but have not yet truly accepted the behavior. In general, extrinsic motivation in academic settings is characterized by pleasing one’s teacher, receiving good grades, or depending heavily on one’s teacher for guidance [39,40]. 

### 2.4. Feedback Engagement

In general terms, a very useful way of organizing conceptualization about engagement in learning in the field of education is the three-category taxonomy of engagement proposed by Fredricks et al. [42]. According to Fredricks et al., student engagement is a multifaceted construct that comprises three categories of learning engagement: cognitive engagement, behavioral engagement, and emotional engagement. Cognitive engagement tends to be conceptualized in terms of psychological investment in learning. Behavioral engagement relates to the time and effort students devoted to their learning, or their participation and persistence in learning activities. The concept of behavioral engagement is usually equated with the more widely used concepts of “persistence”, “effort regulation”, or “effort management” in educational psychology. In fact, this behavioral perspective of engagement is the most widely adopted perspective of student engagement in higher education research, which focuses on the extent to which students become involved in academic and extracurricular activities [43,44]. Emotional engagement refers to students’ positive and negative feelings about their studies, such as interest, anxiety, and boredom. 

In second-language acquisition, Ellis [2] applied the three-category taxonomy of engagement in discussion of feedback processes associated with students’ engagement with oral and written corrective feedback, and highlighted the need to understand the connection between the motive systems and learners’ feedback preferences. Prior research (e.g., [45]) suggests that among the three components of engagement, behavioral engagement best predicts academic learning performance; in line with previous feedback research [46,47], the current study focused on students’ behavioral feedback engagement in the context of learning English as a second language, which was operationalized in the form of (1) *action on teacher feedback*, which was defined in this study as students processing and using the feedback information offed by their teacher, and (2) *feedback seeking*, which was defined in this study as students proactively soliciting information in relation to their learning performance.

In the field of organizational psychology, Ashford and Cummings [48] proposed a model of feedback-seeking behavior (FSB) that defines FSB as the conscious devotion of effort toward obtaining feedback about one’s own performance [49]. The basic tenet of the FSB model is that individuals tend to make a conscious assessment of the values and costs that are associated with the feedback-seeking behavior [49], and such perceptions about the values and costs of feedback seeking are the essential determinants of individuals’ feedback-seeking behavior [50]. For example, it is often suggested that individuals are motivated to seek feedback when it is relevant to their goal pursuing or goal attainment. Tuckey et al. [51] argued that feedback content and seeking may also elicit impression management concerns, suggesting that feedback-seeking behavior can have value as an impression management tool [50]. It is also assumed that individuals’ ego and self-esteem may suffer from the threat of negative feedback, which suggests a cost of exposing one’s uncertainty or insecurity when they risk the potential embarrassment of drawing attention to their performance deficiencies [52]. 

Applying the model of Ashford and Cummings [48] on feedback-seeking behavior in the context of second-language learning, Papi et al. [7] examined the linkage between students’ feedback-seeking behavior and its motivational antecedents (i.e., the learners’ language mindsets and achievement goals). They noted that a growth language mindset and development-approach goals predicted students’ feedback monitoring and feedback inquiry. Using the cost–value framework proposed by Ashford and Cummings, Papi et al.’s interpretation of their results is that as students with a growth mindset and development-approach goals tend to focus on developing rather than validating their L2 abilities, these students are not typically concerned with the ego and self-presentation costs of feedback and monitoring, and perceive obtaining feedback as opportunities for growth and development in the target language. Papi et al. [7] also noted that a fixed language mindset, demonstration approach, and demonstration avoidance predicted feedback inquiry but not feedback monitoring. When interpreting these results, Papi et al. speculated that although students with a fixed mindset and a pursuit of demonstration-approach and demonstration-avoidance goals are concerned with the potential cost associated with feedback seeking to their image, they may either still perceive feedback seeking as being useful to their studies or view feedback seeking as a means of making good impression on their teachers. Additionally, according to Papi et al., these students may seek feedback from their peers and friends, rather than from their teachers, to avoid looking incompetent since peers and friends are not involved in their performance evaluation.

## 3. Methods

### 3.1. Research Context and Participants

The sample comprised 276 second-year university students majoring in English language and literature from two universities that are located in central China. Students are admitted into the English language and literature program based on their assessment results from the National College Entrance Examination. The English language and literature program offers a range of courses, such as English vocabulary and grammar, speaking and writing, and English literature, which are intended to help students develop the understanding, knowledge, and skills they need to use English to communicate effectively in a variety of professional communication contexts. An institution-wide requirement that is worth noting is that students studying in the English language and literature program should take the Test for English Majors—Grade 4 (*TEM-4*) and the Test for English Majors—Grade 8 (*TEM-8*) in their second and fourth year, respectively, in the program. These two English proficiency tests are developed and organized every year by the Chinese Ministry of Education.

The participants in this study were 33 (12%) males and 243 (88%) females. The age of the participants ranged from 17 to 22 years old (two participants did not state their age), with *M*_age_ = 20.46 years and *SD*_age_ = 2.42. In the course of recruiting the participants, we informed the participants that their participation in this study was voluntary and that they could withdraw from the research at any time if they wanted.

### 3.2. Instruments

#### 3.2.1. English Language Self-Efficacy Scale

Items in the English language self-efficacy questionnaire were adapted from [29]. The participants in the current study judged their confidence in different domains of English language proficiency on a 7-point Likert scale, i.e., self-efficacy in speaking (four items, α = 0.90, sample item: “I can participate in a conversation at a normal speed with an English speaker”); self-efficacy in listening (four items, α = 0.86, sample item: “I can understand English films without subtitles”); self-efficacy in reading (four items, α = 0.89, sample item: “I can read English newspapers”); and self-efficacy in writing (four items, α = 0.89, sample item: “I can write a short essay in English on a topic of which I have knowledge”). 

#### 3.2.2. Task Value Scale

Based on Pintrich et al. [53], the task value scale in this study asked the participants to describe how important, interesting, and useful English learning was for them. The Cronbach’s α of the scale in the original study was 0.90. This scale contains six items (e.g., “I am very interested in the content area of this English course”; “I think the English course material is useful for me to learn”; and “It is important for me to learn the material in this English course”). This 7-point Likert scale has demonstrated internal and external reliabilities across numerous studies and samples (e.g., [33]). The task value scale was found to be reliable in this investigation (α = 0.91). 

#### 3.2.3. Intrinsic and Extrinsic Motivation Scale

To assess the participants’ intrinsic and extrinsic motivations in English learning, we modified eight items from Pintrich et al.’s [53] intrinsic and extrinsic orientation scale. In the current study, the 7-point Likert intrinsic scale contains four items (α = 0.85, sample item: “In English class, I prefer course material that arouses my curiosity, even if it is difficult to learn”) that concern the degree to which students perceive themselves to be learning English for intrinsic reasons, such as challenge, mastery, and curiosity. The 7-point Likert extrinsic scale contains four items (α = 0.81, sample item: “Getting a good grade in English class is the most satisfying thing for me right now”) that concern the degree to which students perceive themselves to be learning English for extrinsic reasons, such as grades, rewards, performance, and evaluation by others.

#### 3.2.4. Behavioral Feedback Engagement Scale

As discussed in the earlier section, the behavioral feedback engagement scale used in this study includes two factors: (1) action on teacher feedback and (2) feedback seeking. Items on the two factors were adapted from [54]. The action-on-teacher-feedback factor (6 items, α = 0.91) measured the participants’ tendency to act on or make use of the feedback provided by their teacher. One sample item is “I pay careful attention to feedback on my work and try to understand what it is saying”. The feedback-seeking factor (9 items, α = 0.87) measured the participants’ tendency to generate feedback themselves or seek feedback directly from other people, such as teachers, peers, or other external sources. One sample item is “I ask my teachers to tell me how I can improve my English”. The 7-point Likert behavioral feedback scale was found to be reliable in this investigation (α = 0.87).

### 3.3. Data Analysis

In this study, confirmatory factor analysis (CFA) was first conducted to test the fit to the data to all univariate models that represent the respective constructs. The following model fit indices were used for evaluating the model fit [55]: the chi-square statistic (χ2) and its degrees of freedom (*df*), along with the associated *p*-value; the Comparative Fit Index (CFI; a value equal to or more than 0.90 indicates acceptable model fit); the Tucker–Lewis Index (TLI; a value equal to or more than 0.90 indicates acceptable model fit); the Root Mean Square Error of Approximation (RMSEA; a value less than 0.08 indicates acceptable fit); and the Standardized Root Mean Squared Residual (SRMR) (a value less than 0.08 indicates good fit). Second, descriptive analyses and correlation analyses were conducted. Third, two sets of multiple regression analysis were performed to explore the relative strength of different forms of motivational variables in predicting students’ behavioral feedback engagement in English learning.

## 4. Results

### 4.1. Confirmatory Factor Analysis 

Since each of the instruments used in this study had a clear theoretical lineage, CFA was conducted to examine the construct validity. The CFA results provided evidence of the satisfying model fit of the English language self-efficacy scale, with factor loadings ranging from 0.67 to 0.86: χ2 = 233.28, df = 98; CFI = 0.96; TLI = 0.95; RMSEA = 0.07 (90% CI: 0.059, 0.083); and SRMR = 0.03. The CFA results also indicated that the two-factor motivational scale generally fit the data well: χ2 = 61.70, df = 14; CFI = 0.95; TLI = 0.91; RMSEA = 0.11 (90% CI: 0.084, 0.141); and SRMR = 0.07, with the factor loadings of the items ranging from 0.46 to 0.95. With regard to the task value scale, the CFA results confirmed that the measurement model had a good fit with the data: χ2 = 11.41, df = 6; CFI = 0.99; TLI = 0.99; RMSEA = 0.06 (90% CI: 0.000, 0.108); and SRMR = 0.02, and the factor loadings of the items ranged from 0.63 to 0.89. Finally, with regard to the behavioral feedback engagement scale, two items were deleted because of their strong correlations with other constructs. The CFA results for the remaining 13 feedback behavior items, which factor loadings ranged from 0.52 to 0.88, revealed a good model fit: χ2 = 172.76, df = 57; CFI = 0.96; TLI = 0.94; RMSEA = 0.08 (90% CI: 0.071, 0.101); and SRMR = 0.06.

The Cronbach’s alpha values for the factors shown in Table 1 are 0.92 (English language self-efficacy), 0.85 (intrinsic motivation), 0.81 (extrinsic motivation), 0.91 (task value beliefs), 0.91 (action on teacher feedback), and 0.87 (feedback seeking), indicating the good internal consistency of each scale. Table 1 also shows that the values of CR for the motivational and feedback factors range from 0.75 to 0.95, which markedly exceed the threshold value of 0.60 (Hair et al., 2010). The AVE values of these factors are mostly acceptable, with only two factors being slightly below the threshold value of 0.50 (i.e., AVE = 0.44 for extrinsic motivation; AVE = 0.46 for feedback seeking) [56]. Fornell and Larcker [57] stated that even if the AVE value is less than 0.5, the convergent validity of a construct is still adequate, provided that the composite reliability is higher than 0.6.

### 4.2. Descriptive Statistics and Correlations

The descriptive statistics for the variables and the correlations between these variables are also provided in Table 1. On the sub-scales measuring feedback behavior, the participants appeared to act on teacher feedback with a relatively higher mean score (*M* = 4.82, *SD* = 1.06) than they engaged in feedback seeking (*M* = 4.35, *SD* = 1.01). With regard to the motivational variables, the results show that the highest rating was given to task value beliefs (*M* = 4.79, *SD* = 1.05). This was followed by extrinsic (*M* = 4.49, *SD* = 1.17) and intrinsic motivations (*M* = 4.48, *SD* = 1.15), and then English language self-efficacy (*M* = 4.18, *SD* = 0.95). The students reported roughly identical levels of extrinsic and intrinsic motivations, and their rating of English language self-efficacy was the lowest. The correlation matrix in Table 1 suggests that all the motivational variables have a medium to strong association with action on teacher feedback and feedback seeking, respectively. The highest correlation is between task value beliefs and action on teacher feedback (*r* = 0.70), and the lowest is between extrinsic motivation and action on teacher feedback (*r* = 0.42). Overall, the higher levels of perceived task value, intrinsic and extrinsic motivations, and English language self-efficacy, the more students act on teacher feedback and engage in feedback seeking.

### 4.3. Relations between Motivational Variables and Feedback Engagement

Multiple regression analyses were conducted to investigate the relationships between self-efficacy, task value beliefs, intrinsic and extrinsic motivations, and feedback engagement. As demonstrated in Table 2, with action on teacher feedback as the outcome variable, the model explains 53.1% of the variance, and two motivational variables, task value beliefs (β = 0.404, *p* < 0.001) and intrinsic motivation (β = 0.273, *p* < 0.001), are significant predictors. With *feedback seeking* as the outcome variable, the model accounts for 46% of the variance, and three motivational variables, self-efficacy (β = 0.197, *p* = 0.001), task value beliefs (β = 0.297, *p* < 0.001), and extrinsic motivation (β = 0.185, *p* < 0.001), are significant predictors.

## 5. Discussion

In this study, task value beliefs emerged as the only motivational variable that significantly predicted both action on teacher feedback and feedback seeking. Task value beliefs refer to learners’ evaluation of how important, useful, and interesting a learning task is [58]. The results suggest that students who value learning tasks in English classes are likely to be more motivated to both act upon teacher feedback on their academic work and seek external feedback from their teacher and peers, or by using other means such as a computer. In English learning, when students perceive a subject as being of importance in relation to their future goals, they are willing to study hard and integrate feedback into their learning process [59]. It may be that positive attitudes toward learning tasks serve as a catalyst to stimulate students to directly use the feedback they receive to close the performance gap and to plan the strategies (e.g., seeking external feedback) they may use to improve subsequent work [60]. Consequently, based on the cost–value perspective proposed by Ashford and Cummings [48], it is possible that students who think that English learning is important and useful tend to see much value in acting on teacher feedback and seeking external feedback as they may believe feedback that helps to promote their English learning or performance. For these students, the potential effort costs associated with acting on or seeking feedback are likely dwarfed by the high learning and performance values of feedback [7] This explanation appears to confirm the empirical findings of Papi et al.’s study, which showed that the high value and low cost that students associated with their feedback behavior could lead to their active feedback engagement. 

Intrinsic motivation refers to the situation where the actor is free of coercion, demand, or persuasion to engage in, or not engage in, a particular task [23]. In this study, intrinsic motivation significantly predicted action on teacher feedback. This suggests that intrinsically motivated students may be more likely to act on teacher feedback. This finding aligns with previous research that highlights the role of intrinsic motivation in learner self-regulation through exploring new things and improving themselves [40]. Ryan and Deci [23] argued that intrinsically motivated individuals engage in tasks that interest them, and in doing so, it helps them to learn, develop, and expand their capacities. It may be that when intrinsically motivated students receive teacher feedback as comments on their current work, or as advice for improvement of their future work, they tend to perceive such feedback to be of high learning value, and they are, thus, more likely to act on the feedback information and are more willing to transfer possible feedback insights from one task to another to improve their work and learning strategies. This result echoes a widely shared view in the Confucian heritage culture that Chinese students tend to be characterized by high achievement motivation, hard work, and taking personal responsibility for their learning [61].

The missing link between intrinsic motivation and feedback seeking in this study is somewhat not expected because, according to the self-regulated learning theory, intrinsically motivated students are both persistent and resourceful in that they not only act upon teacher feedback but also actively seek external feedback, for example, from teachers and peer classmates or other external sources [47]. However, as emphasized in previous research, individual learners’ attention to feedback is not an exclusively cognitive mechanism for the intake and processing of feedback; rather, it is a function of personal and situational characteristics [62]. Personal characteristics may include stable motivational characteristics, such as competence-related motives, individual interests, and achievement goals, whereas situational characteristics include task characteristics, such as task difficulty and subject matter, and learning situation characteristics, such as the social setting and the potential gains and losses a learner could anticipate in a situation [63]. Intrinsically motivated learners are usually higher achievers compared with their peer classmates [64]. They may believe that their peer classmates are likely to fail to respond if they seek feedback from them, which in turn will probably threaten their peer classmates’ ego and, therefore, likely taint their image [51]. It has been reported in the literature on second-language acquisition that direct correction of any sort may create a judgmental and, therefore, stressful classroom atmosphere [65]. Given these potential costs, intrinsically motivated learners may sometimes tend to avoid seeking feedback from their peer classmates, particularly in the Chinese cultural context. Therefore, they appear to be more interested in action on teacher feedback but less interested in direct feedback seeking from external sources, such as peer classmates.

In this study, extrinsic motivation had a significant relation with feedback seeking but not with action on feedback. Ashford and Northcraft [64] argued that individuals with performance-prove goals tend to use feedback inquiry to make a positive impression on others as this behavior is likely to help the positive image they try to project so as to satisfy their ego’s need for a sense of superiority. As extrinsic motivation is a construct that is very consistent with the concerns of performance goal-oriented individuals [53], from the perspective of using feedback-seeking behavior for only self-attention or self-enhancement [48], extrinsically motivated individuals may likely perceive a need to seek feedback and may likely engage in a comparably higher frequency of feedback-seeking behavior to highlight their success to others so as to please them [50]. Our results, thus, appear to confirm that students with performance-prove goals are likely to use feedback seeking to make a positive impression on others, as suggested in the literature [7,51,64]. Our study also appears to echo the findings of Lepper et al. [40], who observed that extrinsically motivated students tended to depend heavily on their significant others for guidance. 

Self-efficacy reflects a student’s contextually specific judgments of their abilities or confidence to perform an academic task successfully [66,67]. Research suggests that when individuals feel confident, they are able to learn and are likely to utilize self-regulating processes, such as self-evaluation and self-monitoring (e.g., [68,69]). Therefore, we expected that English-language self-efficacy was a significant predictor of students’ behavioral feedback engagement. Interestingly, our results showed that students with higher self-efficacy had greater tendency to seek feedback than to act on teacher feedback. This result could also be understood with reference to personal and situational characteristics [62]. From both social cognitive and self-determination theoretical perspectives, students with higher self-efficacy tend to pursue a mastery goal orientation, opt for challenging tasks, and cope more effectively with cognitive demands [70,71]. Consequently, it is possible that students with higher self-efficacy have greater tendency to seek feedback than to act on teacher feedback, and they are more likely to put their ideas out for public scrutiny and discuss their thinking and ideas with other people without being concerned with the potential ego, image, and effort costs associated with feedback-seeking behavior [7,67], as they may perceive greater value or learning and competence development opportunity associated with direct feedback seeking. 

## 6. Implications

The present investigation examined the extent to which four motivational variables (i.e., self-efficacy, task value, and *intrinsic* and *extrinsic motivations*) predicted two types of behavioral feedback engagement (i.e., *action on teacher feedback* and *feedback seeking*) in English learning within the context of a tertiary-level English language and literature program. Understanding what keeps students engaged with feedback is of great importance if the inherent power of feedback is to be harnessed [61]. As mentioned earlier, little is known about the mechanisms underlying individual differences regarding how learners’ motivational characteristics impact on their engagement with feedback. The present study addressed this critical gap in the literature and added to our understanding of the dynamics of students’ motivational processes in relation to their feedback engagement in ESL learning settings.

Our key findings demonstrated that the value that the Chinese student participants put on English language learning was an essential positive precursor of feedback engagement. In other words, the students’ perceived instrumentality of English tended to motivate them to engage in feedback action and feedback seeking. Pedagogically, such findings provide important practical implications for teachers on how to motivate students to act on and use feedback. First, ESL classrooms need to be structured in the way in which students are oriented to demonstrate that the learning task is worth pursuing for them in relation to their future. In this regard, when students perceive an ESL learning task to be important and useful, they are likely to exert their best effort to engage in the learning process, of which feedback action and feedback seeking are inextricably a crucial part. After all, as Miller and Brickman point out, “human beings simply do not pursue competence in every area open to them ([65], p. 19). Second, teachers can adopt a learner-centered approach in English teaching, in which facilitation and guidance are provided as the means to accommodate students’ learning needs and styles to promote positive learning attitudes toward English learning among the students. Third, this study also shows that students’ English-language self-efficacy and intrinsic learning orientation significantly predicted their behavioral feedback engagement. Consequently, teachers should provide students with exemplars showing good ways of tackling an assignment, as well as opportunities to rehearse strategies directed at improving English proficiency, to enhance students’ English-language self-efficacy. In order to promote intrinsic motivation to learn, teachers can help students set English learning goals that focus on mastery [70] and provide them with support and conditions for managing challenging English learning situations so that students are likely to have opportunities to experience the development of autonomy and competence.

## 7. Limitations

This study contributes to the literature by providing empirical evidence that feedback is not simply a cognitive process involving only the transfer of information, but feedback is regulated by multiple forms of motivation; however, some limitations to the present study should be acknowledged and direction for future research needs to be provided. First, this investigation was a cross-sectional study and the findings of our study only indicate the associations or relationships between the variables. As such, conclusions about the causal direction of effects, which requires careful experiments, could not be established. In future research, longitudinal design is needed to confirm the direction of the relationships tested. Second, as is the case with other survey-based investigations, this investigation’s use of self-reported data is likely to be subjected to bias. Although self-report has been widely utilized as a valid approach for measuring student perceptions and intra-psychic processes, responses from student self-report might not accurately reflect their true feelings. Future studies could adopt additional objective measures, such as teacher reports or classroom observations, to corroborate the statistical evidence reported in this study. 

Third, this study represents the first attempt to empirically operationalize action on teacher feedback and feedback seeking to investigate their relations to students’ English-language self-efficacy, task value beliefs, and intrinsic and extrinsic motivations. As the items used for measuring these two types of student feedback behavior are relatively newly proposed, further assessment with other populations is needed to provide evidence from external aspects regarding the construct validity of the two scales. Fourth, most of the participants in this study were females as a consequence of the trend across language and literature programs that female students tend to outnumber male students, which limits the generalizability of the current research findings. Fourth, although this study provided insight into the dynamic relationships between motivational processes and student feedback experience in the context of a university-level English language and literature program, the models in this study should be replicated and further examined in other academic subject areas. It is critical to administer the instruments more widely to other languages or other subjects in order to further illuminate the generalizability of the role of motivational factors in students’ feedback engagement across different academic subjects and demonstrate the utility of such instruments. In particular, we suggest that future research replicates this study to examine the generalizability of our findings to different social and cultural contexts and different age or gender groups. Finally, our study is limited in terms of a relatively small sample size. Future studies are encouraged to include a larger sample of students to achieve better generalizability.

## Figures and Tables

**Table 1:** Descriptive statistics, reliabilities, validity, and correlations of the factors.

	M	SD	α	CR	AVE	1	2	3	4	5	6
1. English language self-efficacy	4.18	0.95	0.92	0.95	0.57	1					
2. Intrinsic motivation	4.48	1.15	0.85	0.84	0.59	0.66 **	1				
3. Extrinsic motivation	4.49	1.17	0.81	0.75	0.44	0.39 **	0.45 **	1			
4. Task value beliefs	4.79	1.05	0.91	0.91	0.63	0.59 **	0.70 **	0.49 **	1		
5. Action on teacher feedback	4.82	1.06	0.91	0.92	0.65	0.56 **	0.66 **	0.42 **	0.70 **	1	
6. Feedback seeking	4.35	1.01	0.87	0.86	0.46	0.57 **	0.59 **	0.49 **	0.63 **	0.76 **	1

Note. ** Correlation is significant at the 0.01 level (2-tailed).

**Table 2:** Regression results with self-efficacy, task value beliefs, and motivations as predictors and student feedback behaviors as outcome variables.

Predictors	B	Std. Error	β	t	Sig.	Outcome Variables
Self-efficacy	0.135	0.063	0.119	2.124	0.035	Action on teacher feedback F (4, 267) = 75.659, R^2^ = 0.531, *p* < 0.001
Task value beliefs	0.409	0.062	0.404	6.637	<0.001	
Intrinsic motivation	0.252	0.059	0.273	4.278	<0.001	
Extrinsic motivation	0.039	0.043	0.044	0.907	0.365	
Self-efficacy	0.209	0.064	0.197	3.264	0.001	Feedback seeking F (4, 267) = 56.833, R^2^ = 0.460, *p* < 0.001
Task value beliefs	0.282	0.062	0.297	4.546	<0.001	
Intrinsic motivation	0.138	0.059	0.159	2.312	0.022	
Extrinsic motivation	0.156	0.044	0.185	3.556	<0.001	

## Data Availability

The data presented in this study are available from the corresponding author upon request. The data are not publicly available due to signed consent of the research participants.
